# Bioinformatic analysis of short-chain dehydrogenase/reductase proteins in plant peroxisomes

**DOI:** 10.3389/fpls.2023.1180647

**Published:** 2023-06-09

**Authors:** Yuchan Zhang, Xiaowen Wang, Xinyu Wang, Yukang Wang, Jun Liu, Saisai Wang, Weiran Li, Yijun Jin, Delara Akhter, Jiarong Chen, Jianping Hu, Ronghui Pan

**Affiliations:** ^1^ College of Agriculture and Biotechnology & ZJU-Hangzhou Global Scientific and Technological Innovation Center, Zhejiang University, Hangzhou, China; ^2^ Zhejiang Lab, Hangzhou, China; ^3^ Department of Genetics and Plant Breeding, Sylhet Agricultural University, Sylhet, Bangladesh; ^4^ MSU-DOE Plant Research Laboratory and Plant Biology Department, Michigan State University, East Lansing, MI, United States

**Keywords:** peroxisome, short-chain dehydrogenase/reductase (SDR), peroxisomal targeting signal type 1 (PTS1), fatty acid β-oxidation, benzaldehyde biosynthesis

## Abstract

Peroxisomes are ubiquitous eukaryotic organelles housing not only many important oxidative metabolic reactions, but also some reductive reactions that are less known. Members of the short-chain dehydrogenase/reductase (SDR) superfamily, which are NAD(P)(H)-dependent oxidoreductases, play important roles in plant peroxisomes, including the conversion of indole-3-butyric acid (IBA) to indole-3-acetic acid (IAA), auxiliary β-oxidation of fatty acids, and benzaldehyde production. To further explore the function of this family of proteins in the plant peroxisome, we performed an *in silico* search for peroxisomal SDR proteins from *Arabidopsis* based on the presence of peroxisome targeting signal peptides. A total of 11 proteins were discovered, among which four were experimentally confirmed to be peroxisomal in this study. Phylogenetic analyses showed the presence of peroxisomal SDR proteins in diverse plant species, indicating the functional conservation of this protein family in peroxisomal metabolism. Knowledge about the known peroxisomal SDRs from other species also allowed us to predict the function of plant SDR proteins within the same subgroup. Furthermore, *in silico* gene expression profiling revealed strong expression of most *SDR* genes in floral tissues and during seed germination, suggesting their involvement in reproduction and seed development. Finally, we explored the function of SDRj, a member of a novel subgroup of peroxisomal SDR proteins, by generating and analyzing CRISPR/Cas mutant lines. This work provides a foundation for future research on the biological activities of peroxisomal SDRs to fully understand the redox control of peroxisome functions.

## Introduction

Peroxisomes are ubiquitous single-membrane eukaryotic organelles. Plant peroxisomes participate in many critical metabolic processes, such as β-oxidation of fatty acids, the glyoxylate cycle, photorespiration, H_2_O_2_ detoxification, biosynthesis of phytohormones, and catabolism of sulfite, branched-chain amino acids, urate, and polyamines (Pan et al., 2020). Because peroxisomes lack DNA, peroxisomal proteins are translated in the cytoplasm before being imported into the organelle. Most peroxisomal matrix proteins are equipped with a C-terminal peroxisomal targeting signal type 1 (PTS1) or an N-terminal nonapeptide PTS2, which are recognized and bound in the cytosol by the import receptor PEX5 or PEX7 before the cargo is escorted into the organelle (Baker et al., 2016; Reumann and Chowdhary, 2018). Both targeting pathways are largely conserved in eukaryotes.

About 200 peroxisomal proteins have been identified in the model plant species *Arabidopsis thaliana*, many of which through mass spectrometry (MS)-based peroxisome proteomics studies (Pan and Hu, 2018; Pan et al., 2018). Plant peroxisomes contain many oxidases, such as glycolate oxidase (GOX), acyl-CoA oxidase (ACX), polyamine oxidase (PAO), copper-containing amine oxidase (CuAO), urate oxidase (UOX), and sulfite oxidase (SO), all of which generate H_2_O_2_ as a by-product (Del Río and López-Huertas, 2016; Su et al., 2016). Many other peroxisomal enzymes catalyze redox (reduction-oxidation) reactions such as hydration, dehydrogenation, and reduction. Such examples include the multifunctional proteins AIM1 (abnormal inflorescence meristem) and MFP2 (multi-functional protein 2), each catalyzing two consecutive steps in β-oxidation: hydration and dehydrogenation (Pan and Hu, 2018).

Peroxisomal redox enzymes also include the short-chain dehydrogenase/reductase (SDR) superfamily of NAD(P)(H)-dependent oxidoreductases. Members of this family share three common features: (1) a cofactor binding site (TGxxxGxG) for binding to NAD(P)(H), (2) a catalytic motif YxxxK, and (3) a three-dimensional structure made up of the “Rossmann fold”, which has an α/β folding pattern with a central β sheet flanked by two or three α-helices at each side (Gröger et al., 2012; Moummou et al., 2012). SDR proteins are very diverse in structure and function, only sharing 20–30% amino acid similarity across most family members in the same organism (Kallberg et al., 2009). Plant SDR proteins perform various functions, including fatty acid synthesis and elongation (Ohlrogge and Jaworski, 1997; Beaudoin et al., 2009), chlorophyll degradation (Sato et al., 2009), as well as the metabolism of terpenoids (Okamoto et al., 2011; Czechowski et al., 2022), steroids (Poutanen et al., 1995), phenolics (Wang et al., 2014), and alkaloids (Ziegler et al., 2006). A comprehensive inventory of SDR proteins from 10 plant species identified 178 SDRs from *Arabidopsis* (Moummou et al., 2012).

In *Arabidopsis*, six SDR proteins are known to localize to peroxisomes. These include SDRa/IBR1 (indole-3-butyric acid response 1) (Reumann et al., 2007; Wiszniewski et al., 2009), SDRb/DECR (2,4-Dienoyl-CoA Reductase) (Reumann et al., 2007), SDRc (Reumann et al., 2007; Reumann et al., 2009), SDRd (Quan et al., 2013), SDRe (Pan and Hu, 2018), and NQR (NADH: quinone Reductase) (Quan et al., 2013). SDRa is specifically involved in shortening the side chain of indole-3-butyric acid (IBA) to produce indole acetic acid (IAA) (Wiszniewski et al., 2009). Analysis of SDRc and SDRd knockdown mutant lines suggested that these two SDRs are responsible for benzaldehyde biosynthesis (Huang et al., 2022). The functions of other peroxisomal SDRs in *Arabidopsis* have not been characterized.

To explore the function of plant peroxisomal SDR proteins, we conducted a genome-wide search for genes encoding peroxisomal SDR proteins in *Arabidopsis*, followed by subcellular targeting, phylogenetic, protein structure, expression pattern, and mutant analyses. We identified 10 *Arabidopsis* peroxisomal SDRs, four of which for the first time. Phylogenetic studies of peroxisomal SDRs from diverse species suggested the conserved role of these SDRs in plants and helped us to predict the function of each subgroup based on knowledge of the known peroxisomal SDRs from other species. Moreover, gene expression profiling revealed strong expression of most *SDR* genes in floral tissues and during seed germination, suggesting their involvement in scent production, reproduction and seed development. Finally, we generated loss-of-function mutants of *SDRj*, which represents a new peroxisomal SDR subfamily, to explore its potential function in peroxisomes. This study provides the basis for further and in-depth research to elucidate the diverse functions of SDRs in plant peroxisomes.

## Materials and methods

### Identification of *SDR* genes

The sequences of *SDR* genes and proteins were obtained from the *Arabidopsis* genome databases (TAIR, http://www.arabidopsis.org/). Peroxisomal/putative peroxisomal SDR proteins were determined based on the presence of predicted type 1 or type 2 peroxisome targeting signals (PTS1 or PTS2) (Kunze, 2020; Deng et al., 2022).

### Protein subcellular localization

For tobacco transient protein expression, the coding region of each SDR was first obtained by PCR with *Arabidopsis* cDNA as the template (primers shown in **Table S1**). Fusions between mVenus and the PTS1 peptides were obtained by overlapping PCR as described in (Deng et al., 2022). The PCR product was then cloned into the pCAMBIA1300-YFP vector, which contains the 35S constitutive promoter and had been cut by *Sal*I and *Sac*I (New England Biolabs, Beijing, China), using the ClonExpress II One Step Cloning Kit (Vazyme, Nanjing, China). To generate the peroxisome marker cyan fluorescent protein (CFP or moxCerulean3)-PTS1, an SKL tripeptide was fused to the C-terminus of the CFP or moxCerulean3 before the fusion construct was cloned into the pGWB545 vector backbone (Nakagawa et al., 2007).

The constructs were first transformed into *Agrobacterium tumefaciens* strain GV3101 carrying the helper plasmid pMP90 *via* heat shock (Hofgen and Willmitzer, 1988). Transient protein expression in tobacco (*Nicotiana tabacum*) leaves followed by confocal microscopy to analyze protein targeting was carried out as described previously (Pan et al., 2014). A Fluoview FV3000 confocal laser-scanning microscope (Olympus, Tokyo, Japan) was used for image capturing, where CFP was excited with 445 nm lasers and detected at 460–500 nm and YFP was excited with 514-nm lasers and detected at 530–630 nm.

### Phylogenetic analysis

Homologous peroxisomal SDR proteins from *Physcomitrella patens, Ginkgo biloba, Oryza sativa* and other plants were retrieved by the local blast algorithm and filtrated by checking the presence of PTS1 or PTS2 on the protein sequences. Sequences of known peroxisomal SDR proteins in other species were downloaded from NCBI (https://www.ncbi.nlm.nih.gov/). Phylogenetic analysis of SDRs from different species was conducted using MEGAX (Kumar et al., 2018) by the Jones-Taylor-Thornton (JTT) model.

### Analysis of gene structure and protein motifs

Information about the exon-intron structure of the *SDR* genes was obtained from the genome annotation files downloaded from the *Arabidopsis* (TAIR, http://www.arabidopsis.org/) and rice (Rice Genome Annotation Project, http://rice.uga.edu/) databases. MEME (http://meme.nbcr.net/meme/intro.html) was applied to analyze conserved motifs (Bailey et al., 2015). Results were presented using the TBtools software (Chen et al., 2020).

### Gene expression profiling

The expression values of *Arabidopsis SDR* genes were extracted from the *Arabidopsis* eFP Browser (https://bar.utoronto.ca/efp/cgi-bin/efpWeb.cgi). To compare the expression level of each genes in different tissues or different genes in a specific tissue, z-scores were calculated which is a statistical measurement to describe the relationship of a value to the mean of a group of values.

The formula of z-score is z=(x-μ)/σ. In the formula, z means z-score, x means the value being evaluated, μ means the mean of the group of values and σ means the standard deviation of this group.

In the heatmap, different colors were used to represent the value of the z-score, which indicates the level of gene expression. For the change of *SDRj*’s expression level under different abiotic stress or hormone treatments, log2-fold changes were calculated and displayed between treated conditions and control conditions.

### Plant materials, growth conditions, and phenotypic assays

*Arabidopsis* (*Arabidopsis thaliana*) plants were in the Col-0 background. Sterilized seeds were placed in growth medium containing half-strength Murashige and Skoog medium (1/2 MS) with 0.8% (w/v) agar and 0.5% (w/v) sucrose and stratified at 4°C in the dark for 2 d. After that, plants were transferred to chambers at 23°C under a 16/8h light/dark photoperiod. 10 days later, plants were transferred to soil and grown under the same conditions.

To examine sucrose-dependent hypocotyl elongation in the dark or light, seeds were plated on 1/2MS with or without 1% (w/v) sucrose, grown in the dark or under continuous illumination for 7 d, after which hypocotyl or root lengths were measured.

To examine auxin-responsive root elongation, seedlings were grown under continuous illumination for 7 d on 1/2 MS with 0.8% (w/v) agar and 0.5% (w/v) sucrose supplemented with 0 μM, 8 μM or 16 μM IBA or 0 mg/L, 0.1 mg/L or 0.2 mg/L 2,4-dichlorophenoxybutyric acid (2,4-DB) at 23°C, after which the lengths of primary roots were measured.

To examine propionate and isobutyrate-responsive root elongation, seedlings were grown under a 16/8h light/dark photoperiod for 5 d on 1/2 MS with 0.8% (w/v) agar and 0.5% (w/v) sucrose supplemented with 0 mM, 0.05 mM or 0.1 mM propionate or isobutyrate at 23°C, after which the lengths of primary roots were measured.

### Generation and identification of *Arabidopsis* mutant lines using the CRISPR/Cas9 system

To generate *sdrj* mutants, we employed the CRISPR/Cas9 system for dicot plants, using the same procedure for target sequence selection and vector construction as previously described (Ma and Liu, 2016). The construct was transformed into *Agrobacterium tumefaciens* strain GV3101 (pMP90) *via* heat shock as described (Hofgen and Willmitzer, 1988). Agrobacterium transformation into the Col-0 plants was conducted by floral dip. The transgenic T1 seeds were collected and screened on 1/2 MS medium containing 40 mg/L hygromycin. To identify successful mutations, the fragments covering the mutation sites were amplified from the transgenic lines by PCR and sequenced.

## Results

### Bioinformatic and experimental identification of *Arabidopsis* peroxisomal SDR proteins

**Figure 1:**
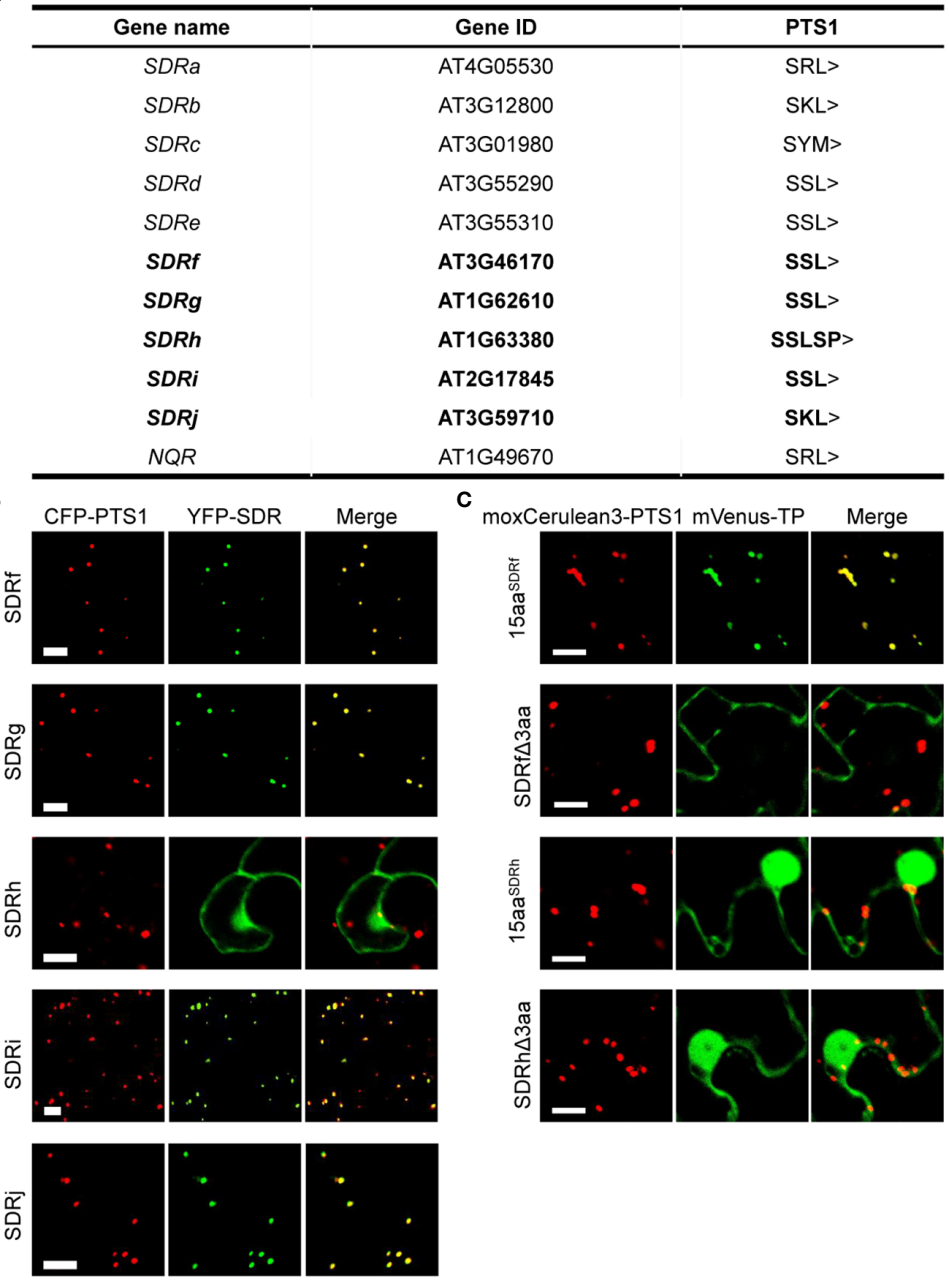
Subcellular localization of peroxisomal SDR proteins newly identified in this study. **(A)** *Arabidopsis* peroxisomal SDR proteins, with the 5 newly identified in bold. **(B)** Confocal microscopy images of the peroxisome localization of YFP-SDRf, YFP-SDRg, YFP-SDRh, YFP-SDRi, and YFP-SDRj in tobacco leaf epidermal cells. Each YFP fusion was co-expressed with the peroxisome marker CFP-PTS1. Scale bars = 10 mm. **(C)** Confocal images of several partial proteins of SDRf and SDRh in tobacco leaf epidermal cells. 15aa indicates the last 15 amino acids of the protein. Δ3aa indicates the protein without the last 3 amino acids. Each mVenus fusion was co-expressed with the peroxisome marker moxCerulean3-PTS1. Scale bars = 10 mm.

A previous systematic study of the plant SDR superfamily identified 178 SDRs from the *Arabidopsis* genome (Moummou et al., 2012). To identify peroxisomal SDRs, we downloaded protein sequences of these 178 SDRs from TAIR (http://www.arabidopsis.org/) and manually searched for PTS1 and PTS2 peptides in the proteins (Kunze, 2020; Deng et al., 2022). A total of 11 proteins were found to contain C-terminal PTS1 or PTS1-like sequences, among them 6 are known peroxisomal proteins. Proteins encoded by AT3G59710, At3g46170, At1g62610, At2g17845, and AT1G63380 had not been experimentally determined to be peroxisomal (**Figure 1A**). We named these 5 new SDR proteins SDRf (AT3G46170), SDRg (AT1G62610), SDRh (AT1G63380), SDRi (AT2G17845) and SDRj (AT3G59710) (**Figure 1A**). To check the existence of potential N-terminal signal peptides, we analyzed these SDR proteins using DeepLoc (https://services.healthtech.dtu.dk/services/DeepLoc-2.0/) (Thumuluri et al., 2022) and TargetP (https://services.healthtech.dtu.dk/services/TargetP-2.0/) (Armenteros et al., 2019), which did not find any signals other than the peroxisomal signal.

We then analyzed the subcellular localization of these 5 SDR proteins, which contain three different types of PTS1-like peptides. Among them, SDRj contains SKL>, three (SDRf, SDRg and SDRi) end with SSL>, and SDRh ends with SSLSP>, which does not meet with the composition of a normal PTS1 peptide. We fused the coding sequence of each protein to the C-terminus of yellow fluorescent protein (YFP) and transiently expressed the fusion constructs in tobacco leaves. Co-expressed with each construct was the peroxisome marker containing a fusion of cyan fluorescent protein and the PTS1 tripeptide SKL (CFP-PTS1). SKL> is a canonical PTS1 tripeptide, which usually fits with the consensus sequence of [SA][KR][LM]> (Tarafdar and Chowdhary, 2022). YFP fusions of SDRf, SDRg, SDRi, and SDRj, each containing a canonical PTS1 tripeptide, localized specifically to peroxisomes (**Figure 1B**). YFP-SDRh, which possesses two extra amino acids after the C-terminal tripeptide (SSLSP>), localized to multiple subcellular compartments, including the nucleus, and the cytosol, but not peroxisomes (**Figure 1B**). Since SKL> is the most canonical PTS1 peptide, we did not further analyze it. Instead, we chose to carefully analyze the PTS1 peptide of SDRf (with SSL>) and SDRh (with SSLSP>), including two truncated versions, one without the last 3 amino acids and one containing only the last 15 amino acids. We found that the C-terminal 15aa of SDRf can well localize to peroxisomes, but not when its C-terminal 3aa was deleted. Hence, SDRf does end with a PTS1 peptide. In contrast, none of the two versions of SDRh could localize to peroxisomes, indicating it is not a peroxisomal protein and its C-terminal SSLSP> is not a functional PTS1 (**Figure 1C**).

### Phylogenetic analysis of plant peroxisomal SDR proteins suggested functional diversification between subgroups

The identification of 10 peroxisome-localized SDR proteins from *Arabidopsis* suggested the potential diverse role of this protein family in peroxisomes. To determine how peroxisomal SDR proteins have evolved in plants, we used the 10 *Arabidopsis* SDR protein sequences to perform blast searches against the proteome databases of the bryophyte *Physcomitrella patens*, the gymnosperm *Ginkgo biloba*, and the monocot angiosperm *Oryza sativa*. A total of 16 sequences, all of which contain PTS1, were retrieved and used to construct a Maximum Likelihood tree. To help to predict the function of peroxisomal SDR proteins, SDRh and a number of peroxisomal or non-peroxisomal SDR proteins from various species with known biochemical functions were also included in the phylogenetic analysis.

**Figure 2:**
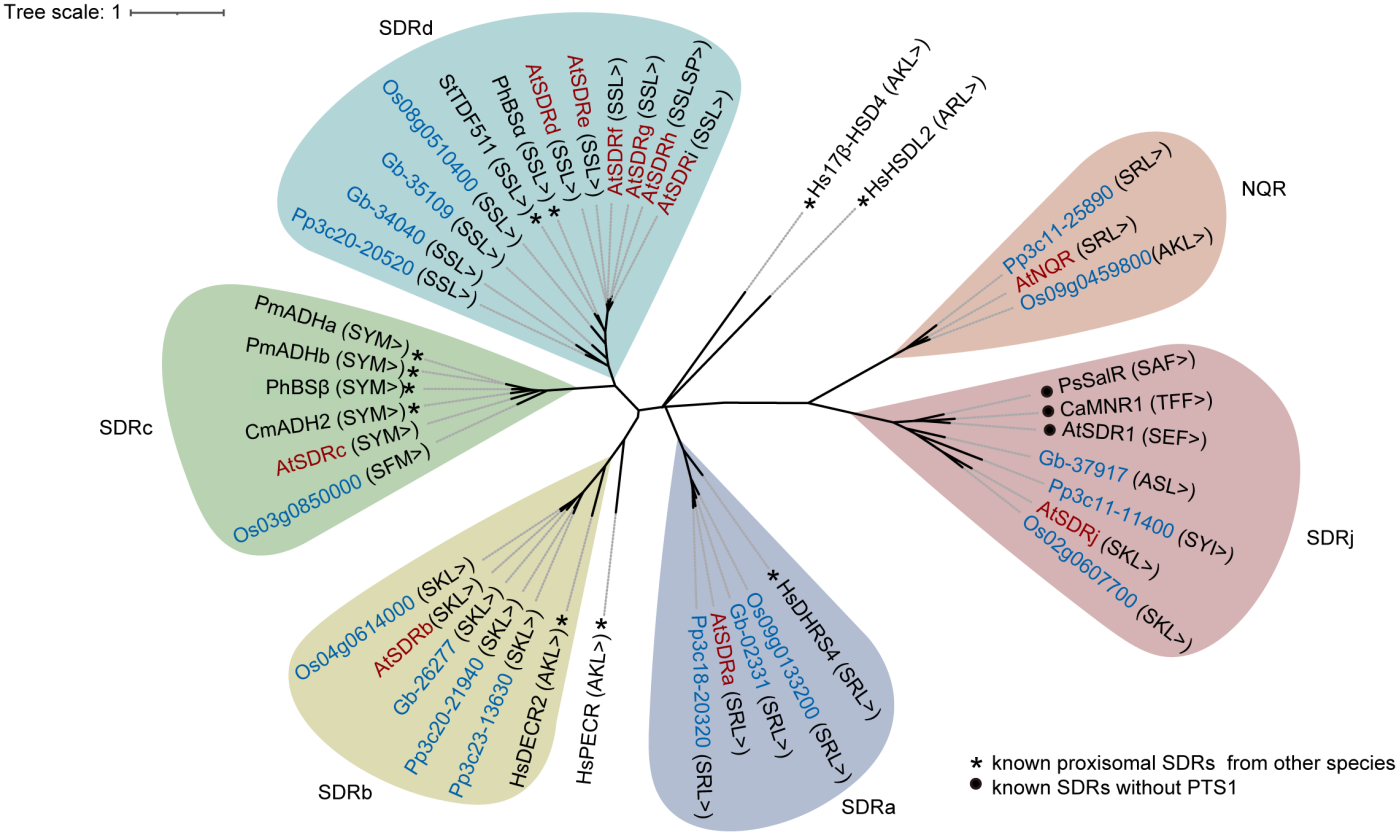
Phylogenetic analysis of SDR proteins in *Arabidopsis thaliana* (At) (red words) and their homologs (blue words) in *Physcomitrella patens* (Pp), *Ginkgo biloba* (Gb) and *Oryza sativa* (Os). Besides of the *Arabidopsis* peroxisomal SDRs involved in this study, previously reported peroxisomal SDR proteins in human and plants (*) were included for functional reference. Several non-peroxisomal but functionally established SDR proteins (●) showing high sequence similarity with SDRj were also included in the phylogenetic tree for functional reference of SDRj. The phylogenetic tree was constructed using MEGAX (Kumar et al., 2018) by Maximum Likelihood method and the Jones-Taylor-Thornton (JTT) model. Ca, *Capsicum annuum*; Cm: *Cucumis melo*; Hs: *Homo sapiens*; Ph: *Petunia hybrida*; Pm: *Persicaria minor*; Ps: *Papaver somniferum*; St: *Solanum tuberosum*.

We first checked the phylogenetic relation between *Arabidopsis* SDRs and those from other plant species. Eight of the 10 *Arabidopsis* peroxisomal SDRs have sequence homologs in *Physcomitrella patens*, *Ginkgo biloba* and rice, except for SDRc, which has a putative peroxisomal homolog in rice but not the other two genomes, and NQR, which does not have an obvious peroxisomal homology in gymnosperms (**Figure 2**). Moreover, SDRc is also grouped with homologs from *Cucumis melo*, *Petunia hybrida*, and *Persicaria minor*, indicating its conservation across angiosperms. In general, these SDRs clustered into 6 subgroups based on the relative distance in the phylogenetic tree (**Figure 2**), which is consistent with the classification described in the previous study (Moummou et al., 2012), indicating that these proteins are likely peroxisome-localized SDRs shared among land plants.

We then further checked the phylogenetic relation between Arabidopsis SDRs with SDRs from other non-plant species. First, SDRa is grouped with human DHRS4 (Dehydrogenase/Reductase 4) (**Figure 2**), which is a peroxisomal protein (Matsunaga et al., 2008; Endo et al., 2009). Second, SDRb is grouped with the mammalian peroxisomal 2,4-dienoyl-CoA reductase (DECR), an auxiliary β-oxidation enzyme (He et al., 1995; Gurvitz et al., 1997; Fransen et al., 1999; Hua et al., 2012), and more distantly related to the human peroxisomal trans-2-enoyl-CoA reductase (PECR) (Gloerich et al., 2006) (**Figure 2**), which belongs to another subfamily (Persson and Kallberg, 2013). Third, SDRc, SDRd, SDRe, SDRf, SDRg, SDRi, SDRj and NQR are not grouped with any non-plant SDRs with established functions (**Figure 2**), indicating that they are most likely involved in plant-specific functions, such as secondary metabolic functions. Finally, two human peroxisomal SDRs, HsHSDL2, Hs17β-HSD4, cannot be grouped with any of the *Arabidopsis* SDRs (**Figure 2**), showing the diversification of SDR functions in different species (Möller et al., 1999; Breitling et al., 2001; Skogsberg et al., 2008; Kowalik et al., 2009; Han et al., 2021).

### Gene structure and protein conserved motif analysis of *Arabidopsis* and rice SDRs support functional conservation within each subgroup

**Figure 3:**
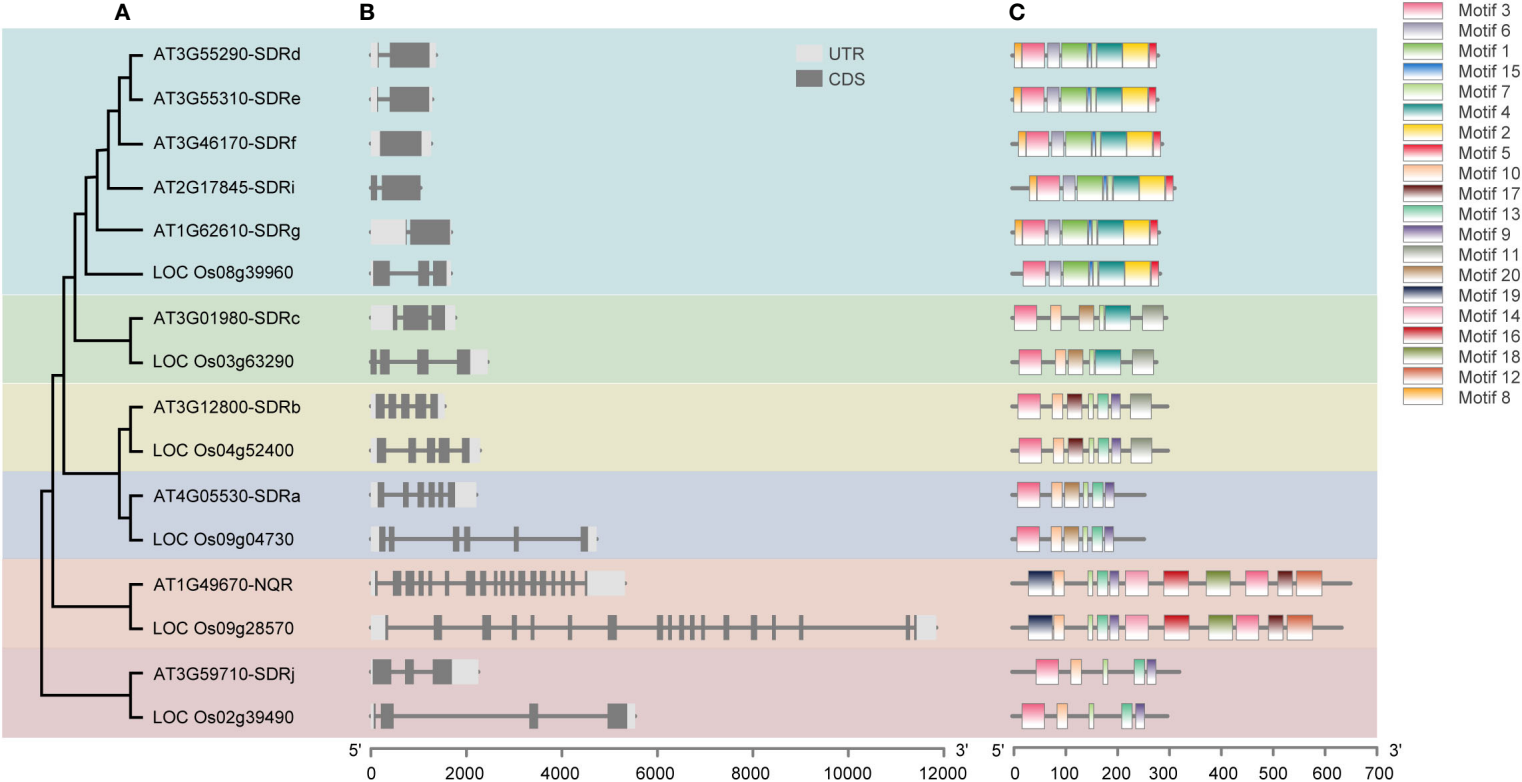
Analysis of the phylogenetic relationship **(A)**, gene structure **(B)** and conserved motifs **(C)** of peroxisomal *SDR* genes in *Arabidopsis* and their orthologs from rice. The maximum likelihood (ML) tree under the Jones-Taylor-Thornton (JTT) model was constructed using MEGAX and full-length protein sequences. The exon-intron structures of these genes were graphically displayed by Tbtools using the CDS and genome sequence of the SDRs. The MEME Suite web server (http://meme.nbcr.net/meme/intro.html) was used to predict conserved motifs in the proteins.

We then analyzed the gene structures and protein motifs for *Arabidopsis* and rice peroxisomal SDRs, using the TBtools software and the online tool MEME (http://meme.nbcr.net/meme/intro.html). Consistent with the results of the sequence-based phylogenetic analysis (**Figure 2**), conserved exon-intron organization and similar patterns of protein motif distribution were found in closely related genes/proteins in the same subgroup (**Figure 3**). The SDR superfamily encodes a large number of enzymes with a broad spectrum of metabolic functions. The similar gene structures and conserved motif distributions displayed in the same subgroups supports the view that SDR proteins in the same clade perform similar functions.

### *In silico* expression profiling of *Arabidopsis* peroxisomal *SDR* genes suggested a key role for these enzymes in reproduction and seed germination

**Figure 4:**
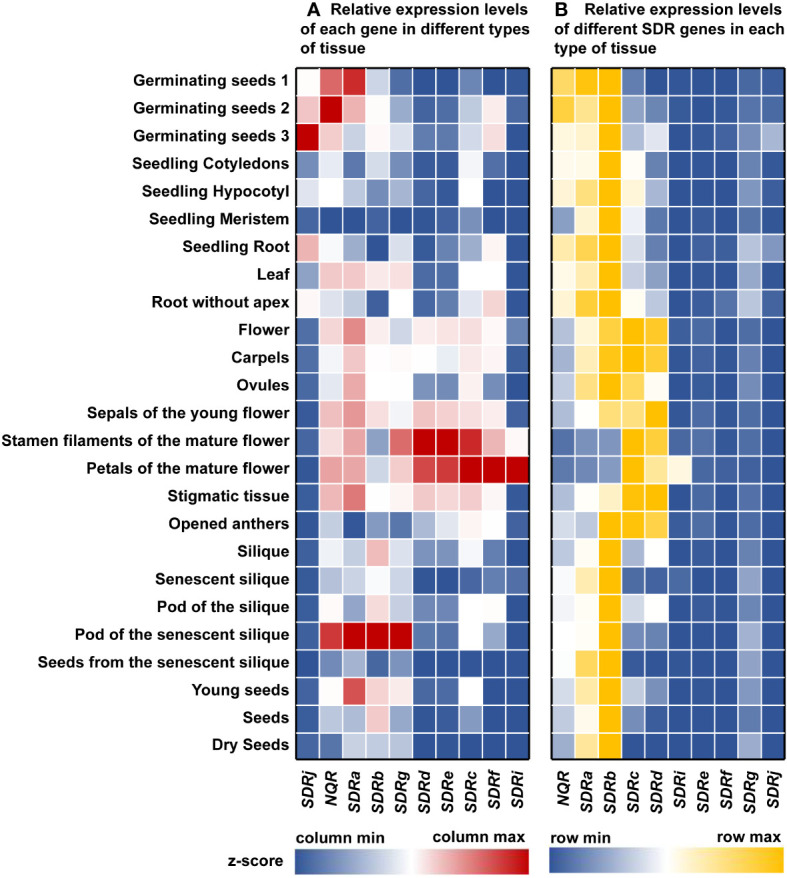
Gene expression profile analysis of *Arabidopsis* peroxisomal *SDR* genes in various tissues. The expression data of *Arabidopsis SDRs* were collected from the *Arabidopsis* eFP Browser (https://bar.utoronto.ca/efp/cgi-bin/efpWeb.cgi). **(A)** Relative expression levels of each gene in different types of tissue. Relative expression data of each gene within each column was shown as z-score. **(B)** Relative expression levels of different *SDR* genes in each type of tissue. Relative expression data of different genes the same tissue type within each row was shown as z-score. z-score was calculated as described in Materials and Methods. Red or yellow represents relatively high expression, blue represents relatively low expression, and white represents the average expression level.

To better understand the role of the peroxisomal SDRs in plants, we analyzed the expression profile of *Arabidopsis* genes encoding peroxisomal SDRs, using expression data from the *Arabidopsis* eFP Browser (Klepikova et al., 2016). *SDRi*, *SDRf*, *SDRc*, *SDRe*, and *SDRd* have the highest expression levels in mature flowers, such as petals, stamen filaments, and sepals, and *SDRg, SDRb, SDRa* and *NQR* are highly expressed in the pods of senescence siliques (**Figure 4A**). The strong expression of these genes in reproductive organs suggests that they may be involved in reproductive processes. *SDRj* and *NQR* show the highest level of expression in germination seeds, displaying a pattern of gradual increase during germination (**Figure 4A**). Besides pods of the senescent silique, *SDRa* is also relatively high expressed in young seeds, floral tissues, and germination seeds (**Figure 4A**).

The expression patterns may help us to predict the function of some plant peroxisomal SDRs. For example, *Arabidopsis SDRc*, *SDRd*, *SDRe*, *SDRf*, *SDRg* and *SDRi* all have relatively high expression levels in floral organs, which is consistent with the role of SDRc and SDRd in the formation of benzaldehyde, an important component in floral scent (Huang et al., 2022). Since SDRe, SDRf, SDRg, and SDRi are close homologs of SDRd, we predict that these proteins may play similar biochemical functions in benzaldehyde production (**Figure 2**).

We also compared the expression levels of different *Arabidopsis* peroxisomal *SDR* genes in the same tissue, and found that *NQR*, *SDRa*, *SDRb*, *SDRc*, and *SDRd* are expressed at relatively higher level in most of the time, while the expression of other *SDR* genes are relatively low (**Figure 4B**). The fact that many *SDRs* are expressed in floral tissues led us to speculate that some of these proteins play a role in the benzenoids biosynthetic pathway during flower development. In germinating seeds, these *SDRs* may be involved in fatty acid β-oxidation.

### Generation and analysis of the *Arabidopsis sdrj* mutants

**Figure 5:**
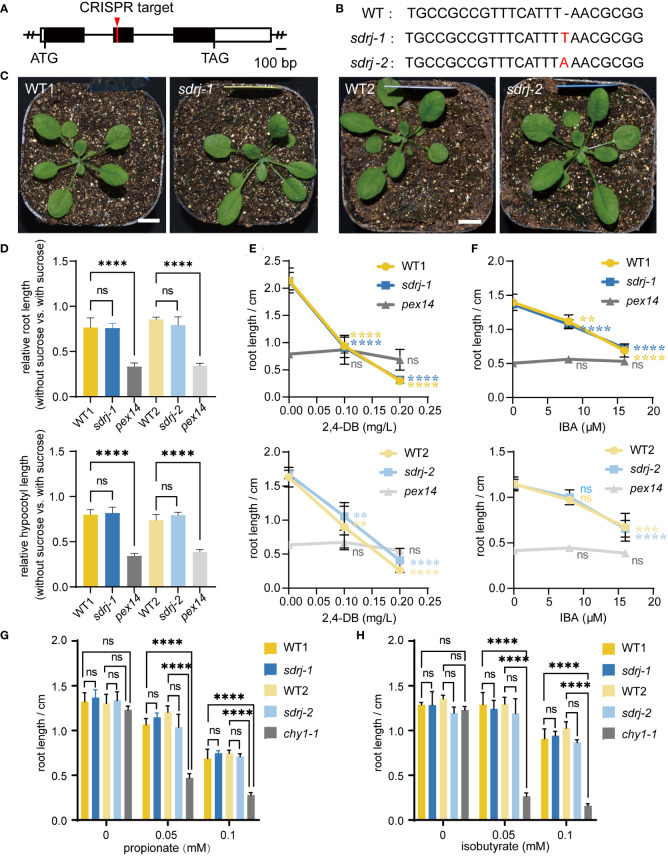
Generation and characterization of the *sdrj* mutants. **(A)** *SDRj* gene structure. Open box, UTR; black box, exon; solid line, intron. CRISPR target site is indicated. **(B)** Mutation sites in the *sdrj* mutants. A 1-bp insertion was found in the second exon in both mutants. **(C)** Appearance of 3-week-old plants. Bar = 1 cm. **(D)** Sucrose dependence assays. For each mutant, all data were normalized to the data in sucrose-containing media. Error bars indicate standard deviations (n=4, the average length of more than 8 seedlings was used as one biological replicate). **** means P < 0.0001 from WT1 or WT2, and ns means no significant difference from WT1 or WT2, as determined by one-way ANOVA. **(E–H)** 2,4-DB, IBA, propionate and isobutyrate response assays. Root lengths on medium containing different concentration of 2,4-DB, IBA, propionate and isobutyrate are shown. Error bars indicate standard deviations (n=4, the average length of more than 12 seedlings was used as one biological replicate). **** means P < 0.0001, *** means P < 0.001, ** means P < 0.01, from 0 concentration, and ns means no significant difference from 0 concentration, as determined by one-way ANOVA.

Unlike other peroxisomal SDRs, SDRj is in a subgroup that does not contain any known peroxisomal proteins and therefore its function cannot be easily predicted. To explore the physiological function of SDRj, we constructed two *sdrj* mutants using the CRISPR/Cas9 technology (**Figure 5A**). By sequencing the cDNA of *SDRj*, it showed that one thymine and one adenine were inserted in the second exon of *SDRj* in *sdrj-1* and *sdrj-2*, respectively, both resulting in frame shift (**Figure 5B**). No obvious growth defects were observed in these *sdrj* mutants under a 16/8 h light/dark photoperiod (**Figure 5C**).

Given *SDRj*’s highest expression during seed germination (**Figure 4**), we speculated that SDRj may be involved in fatty acid β-oxidation which is one of the major peroxisomal function in this period. To determine this hypothesis, the mutants were subjected to sucrose dependence, IBA response, and 2,4-DB response assays. This is because disruption of β-oxidation can impair fatty acid degradation and seed storage oil mobilization, and hence the β-oxidation mutants depend on exogenous sucrose for root and hypocotyl growth. Moreover, disruption of β-oxidation can impair IBA conversion to IAA or 2,4-DB conversion to 2,4-D, which leads to IBA and 2,4-DB resistance in the β-oxidation mutants. The mutant of *pex14* (SALK_007441) is a peroxisome biogenesis mutant with severe β-oxidation defects and was used as a positive control of β-oxidation defective mutants. In sucrose-free medium, *pex14* roots and hypocotyls were much shorter than wild type, but *sdrj* mutants appeared similar to wild-type plants irrespective of the existence of exogenous sucrose (**Figure 5D**). In addition, the root length of the *pex14* mutant showed resistance to different concentrations of IBA or 2,4-DB (**Figures 5E, F**). However, *sdrj* mutants as well as wild-type plants showed similar sensibility to IBA and 2,4-DB (**Figures 5E, F**). Therefore, we concluded that SDRj may not play a significant role in β-oxidation.

In addition, the mutants of the peroxisomal CHY1 is known to be sensitive to propionate and isobutyrate. To check whether SDRj is involved in propionate and isobutyrate metabolism, we treated *sdrj* mutants with propionate and isobutyrate and used *chy1* as the positive control, we found that *chy1-1* (SALK_025417) mutant displayed obvious sensitivity to propionate and isobutyrate in comparison with the wild type (**Figures 5G, H**). In contrast, the *sdrj* mutant exhibited the same level of sensitivity with the wild type (**Figures 5G, H**). These data indicate that SDRj is not necessary for propionate and isobutyrate metabolism.

Since we did not find an obvious role of SDRj in the physiological analysis of its mutants. We performed further analyses on its evolution and gene expression. First, we checked potential SDRj homologs through BLAST search for best-match proteins followed by phylogenetic analysis. Only in embryophytes, but not in green algae, could we find SDRj homologs containing potentially functional PTS1 (canonical PTS1 tripeptide SKL> in *Thuja plicata*, *Amborella trichopoda*, *Oryza sativa*, *Zea mays* and *Solanum lycopersicum*, non-canonical PTS1 tripeptides ASL> in *Ginkgo biloba* and SYI> in *Physcomitrella patens*) (see **Figure S1**). Second, we checked the gene expression of *SDRj* during various hormone and abiotic stress treatments according to the data in (https://bar.utoronto.ca/efp/cgi-bin/efpWeb.cgi), which showed that it could be most obviously induced by Methyl Jasmonate (MeJA) (see **Figure S2**), indicating a potential functional relation with JA. Third, we checked the co-expressed genes with *SDRj* using ATTED-II website (https://atted.jp/gene_coexpression/?gene_id=825140&sp=ath). Among the 2000 co-expressed genes, we could see 31 genes encoding known peroxisomal proteins, such as *Acyl-CoA Oxidase 1* (*ACX1*) and *12-Oxo-Phytodienoic acid Reductase 3* (*OPR3*) (see **Table S1**) that are involved in JA biosynthesis. Hence, further experimental exploration of this protein may focus on its functional relation with JA in the future.

## Discussion

SDR enzymes play critical roles in various physiological and metabolic processes from archaea and bacteria to eukaryotes in different cellular compartments, including peroxisomes, which are dynamic organelles housing rich critical oxidative metabolic reactions. Thus far, genome wide identification of the SDR superfamily has been done in several species, including plants (Kallberg et al., 2009; Moummou et al., 2012; Yu et al., 2021), cyanobacteria (Kramm et al., 2012), human (Bray et al., 2009; Kallberg et al., 2009), nematode and fruit fly (Kallberg et al., 2009), Nile Tilapia (Zhang et al., 2021). However, the genome-wide evolution, expression, and function of the SDR superfamily in the plant kingdom, especially in peroxisomes, have yet to be elucidated clearly. Overall identification and expression analyses of the peroxisomal SDR members are of great importance to define their diverse biological functions in plant peroxisomes.

In this study, we identified ten Arabidopsis peroxisomal SDR superfamily proteins and confirmed the peroxisomal localization of four of them for the first time. Also, we performed phylogenetic analyses of these proteins, their peroxisomal homologs from three representative plants, *Physcomitrella patens*, *Ginkgo biloba* and *Oryza sativa*, as well as some known SDRs from other plant and non-plant species. We also analyzed the gene structure and protein conserved motif of rice and Arabidopsis peroxisomal SDR proteins. These *in silico* analysis supports the functional conservation of SDR proteins within the same subgroup and their diversification across different subgroups. We also analyzed the gene expression pattern of Arabidopsis *SDRs* to predict their physiological functions and generated and experimentally analyzed the mutants of *SDRj*, a novel peroxisomal *SDR* with no known peroxisomal homologs in other species.

SDR superfamily encoded a large number of enzymes and displayed a broad spectrum of metabolic functions. Six subgroups of Arabidopsis peroxisomal SDR proteins were identified based on their relative distance in our phylogenetic tree analysis (**Figure 2**). A recent study found that proteins from different species in the same subgroup of PhBSα have similar enzymatic activity (Huang et al., 2022). Therefore, analyzing the substrates and functions of the same family of proteins is likely helpful for understanding unknown proteins, though the functional similarity of proteins in the same subgroup still needs experimental verification. Among six known Arabidopsis peroxisomal SDRs, SDRa is also named IBR1, as its loss-of-function was identified in an EMS mutant screen for being resistant to IBA, it functions in the conversion of IBA to IAA (Zolman et al., 2008); the mutants show defects in IBA conversion to IAA but not in fatty acid degradation (Zolman et al., 2007; Wiszniewski et al., 2009). In the phylogenetic tree, SDRa is grouped with the peroxisome-localized human protein DHRS4 (**Figure 2**), which acts as retinol dehydrogenase and reductase (Lei et al., 2003) and metabolizes several aromatic carbonyl compounds, steroids, and bile acids (Matsunaga et al., 2008; Endo et al., 2009). The functional mechanism of SDRa is still unknown. However, as suggested by the functions of DHRS4, it may reduce a carbonyl group during IBA to IAA conversion.

With high sequence similarity, SDRb is grouped with mammalian and yeast peroxisome-localized 2,4-dienoyl-CoA reductases (DECRs) (**Figure 2**), it is a strong candidate for DECR activity in plant peroxisomes (Pan and Hu, 2018). DECR is an auxiliary enzyme in the β-oxidation of unsaturated fatty acids, catalyzing the NADPH-dependent reduction of Δ2, Δ4-dienoyl-CoA esters to trans-3-enoyl-CoA esters (He et al., 1995; Gurvitz et al., 1997; Fransen et al., 1999; Hua et al., 2012). More distantly related to SDRb in this group is the human peroxisomal trans-2-enoyl-CoA reductase (PECR) (Gloerich et al., 2006) (**Figure 2**), an enzyme proposed to play a key role in fatty acid chain elongation (Das et al., 2000) besides its demonstrated function in mediating the conversion of phytol to phytanoyl-CoA (Gloerich et al., 2006).

Analysis of the SDRc and SDRd knockdown mutant lines suggested that these two SDRs are responsible for benzaldehyde biosynthesis in Arabidopsis and exhibited strict substrate specificity towards benzoyl-CoA, similar to their homologues PhBSβ and PhBSα from Petunia hybrida (Huang et al., 2022). Although it does not have apparent homologs in the moss *Physcomitrella patens* and the gymnosperm *Ginkgo biloba*, SDRc is grouped with several PTS1-containing SDRs from higher plants, including CmADH2 from *Cucumis melo* (Manríquez et al., 2006; Jin et al., 2016), PmADHa and PmADHb from *Persicaria minor* (Hamid et al., 2018), and PhBSβ from *Petunia hybrida* (Huang et al., 2022) (**Figure 2**). CmADH2 is a short-chain alcohol dehydrogenase with higher activity in alcohol reduction than oxidation and strong substrate specificity towards acetaldehyde, and was predicted to use NADH as a co-factor (Manríquez et al., 2006). The expression of *PmADHa* and *PmADHb* are up-regulated by ABA treatment and drought stress, suggesting their probable role in ABA signaling and drought response (Hamid et al., 2018). PhBSβ is a subunit of a heterodimeric enzyme PhBS, a benzaldehyde synthase with strict substrate selectivity for benzoyl-CoA (Huang et al., 2022).

Interestingly, there are four Arabidopsis SDRd homologs in one subfamily (**Figure 2**), all of which share a high degree of residue identity (78.62-96.43%) and are highly expressed in stamen filaments or petals (**Figure 4**), suggesting their functions are likely to be at least partially redundant. This subfamily includes PhBSα, a subunit of PhBS for benzaldehyde biosynthesis from *Petunia hybrida* (Huang et al., 2022), and also includes StTDF511 from *Solanum tuberosum* (Bachem et al., 2001) (**Figure 2**). The StTDF511 protein was suggested to be involved in the metabolism of a steroid-like compound that affects GA levels in the plant (Bachem et al., 2001). Still, we cannot exclude the possibility that proteins in the same subfamily may have distinct functions.

In this study, we found that SDRj do not play an important function in β-oxidation and the metabolism of propionate and isobutyrate (**Figure 5**). The lack of any obvious defects of *sdrj* in these experiments can be caused by different reasons. For example, we probably did not use the right enzymatic substrates to treat the mutants. Or other peroxisomal SDR proteins may have redundant functions with SDRj. Moreover, since the *sdrj* mutation site is in the middle of the protein, the N-terminal half of SDRj may still have some residual function.

The similar phenotype between *sdrj* mutant and wild type means the function of SDRj needs further investigation. SDRj is grouped with several plant menthone reductases involved in monoterpene metabolism and salutaridine reductases in alkaloid metabolism, none of which is peroxisomal (**Figure 2**). These enzymes include the *Capsicum annuum* menthone: (+)-(3S)-neomenthol reductase (CaMNR1) and its *Arabidopsis* ortholog SDR1 (At3g61220) (Hyong et al., 2008), and *Papaver somniferum* salutaridine reductase (PsSalR) (Ziegler et al., 2006) (**Figure 2**). CaMNR1 and AtSDR1 exhibit enzymatic activities in menthone reduction and are involved in plant defense against both bacterial and fungal pathogens (Hyong et al., 2008). PsSalR is specific for the production of morphinan alkaloids, reducing the keto group of salutaridine to yield salutaridinol, an intermediate in morphine biosynthesis (Ziegler et al., 2006). In consideration of the function of these three proteins (CaMNR1, AtSDR1 and PsSalR) in the same subgroup as SDRj, we reasoned that SDRj may prefer a substrate containing a carbonyl group though the enzyme activity and substrate for SDRj is not clear and needs further explorations. In addition, since SDRj’s PTS1 signal is likely evolved and conserved in land plants, it may have a role in plant adaption to land environment (**Figure S1**). Also, *SDRj*’s expression is induced by MeJA (**Figure S2**) and co-express with some proteins involved in JA biosynthesis (**Table S1**), it may have a functional relation with JA. Future study of SDRj shall focus on these aspects.

NQR is a putative NADPH: quinone reductase involved in plant stress response (Babiychuk et al., 1995), which was first discovered by peroxisome proteome analysis and confirmed by *in vivo* targeting analysis (Eubel et al., 2008).

In peroxisomal SDR family with known function, there are two human peroxisomal SDRs, HsHSDL2, and Hs17β-HSD4, which cannot be grouped with any of the *Arabidopsis* SDRs (**Figure 2**). Hydroxysteroid dehydrogenase-like 2 (HSDL2), which contains an N-terminal SDR domain and a C-terminal Sterol carrier protein 2-like (SCP2-like) domain (Kowalik et al., 2009), plays an important role in fatty acid metabolism (Skogsberg et al., 2008; Han et al., 2021). 17β-HSD4, who was also named multifunctional protein 2 (MFP-2) (Moeller and Adamski, 2006), contains three functionally distinct domains: the N-terminal region contains activities of 17β-estradiol dehydrogenase type IV and D-specific 3-hydroxyacyl CoA dehydrogenase, the middle region has D-specific hydratase activity with straight and 2-methyl-branched 2-enoyl-CoAs, and the C-terminus is identical to SCP2 (Möller et al., 1999). This enzyme mainly functions in the peroxisomal β-oxidation of fatty acid metabolism and has a possible minor role in inactivating estradiol by converting it to estrone (Breitling et al., 2001). The deficiency of this protein causes a very severe Zellweger-like phenotype in human (Möller et al., 2001). It is possible that these two human peroxisomal SDRs arose during the evolution of mammals.

## Data availability statement

Publicly available datasets were analyzed in this study. This data can be found here: GenBank assembly accession: GCA_000001735.2, GCA_001433935.1.

## Author contributions

RP conceptualized and supervised the study. YZ, YW, SW, JL, and WL performed the localization analysis. YZ, YJ, and DA performed the bioinformatic analysis. YZ, XWW, XYW and JC performed the mutant analysis. JH participated in project discussions. YZ, JH, and RP co-wrote the manuscript and generated the figures. All authors contributed to the article and approved the submitted version.

## References

[B1] Armenteros J. J. A. Salvatore M. Emanuelsson O. Winther O. Von Heijne G. Elofsson A.. (2019). Detecting sequence signals in targeting peptides using deep learning. Life Sci. Alliance 2, 1–14. doi: 10.26508/lsa.201900429 PMC6769257 31570514

[B2] Babiychuk E. Kushnir S. Belles-Boix E. Van Montagu M. Inze D. (1995). Arabidopsis thaliana NADPH oxidoreductase homologs confer tolerance of yeasts toward the thiol-oxidizing drug diamide. J. Biol. Chem. 270, 26224–26231. doi: 10.1074/jbc.270.44.26224 7592828

[B3] Bachem C. W. B. Horvath B. Trindade L. Claassens M. Davelaar E. Jordi W.. (2001). A potato tuber-expressed mRNA with homology to steroid dehydrogenases affects gibberellin levels and plant development. Plant J. 25, 595–604. doi: 10.1046/j.1365-313X.2001.00993.x 11319027

[B4] Bailey T. L. Johnson J. Grant C. E. Noble W. S. (2015). The MEME suite. Nucleic Acids Res. 43, W39–W49. doi: 10.1093/nar/gkv416 25953851 PMC4489269

[B5] Baker A. Hogg T. L. Warriner S. L. (2016). Peroxisome protein import: a complex journey. Biochem. Soc Trans. 44, 783–789. doi: 10.1042/BST20160036 27284042 PMC4900764

[B6] Beaudoin F. Wu X. Li F. Haslam R. P. Markham J. E. Zheng H.. (2009). Functional characterization of the arabidopsis β-ketoacyl-coenzyme a reductase candidates of the fatty acid elongase. Plant Physiol. 150, 1174–1191. doi: 10.1104/pp.109.137497 19439572 PMC2705042

[B7] Bray J. E. Marsden B. D. Oppermann U. (2009). The human short-chain dehydrogenase/reductase (SDR) superfamily: a bioinformatics summary. Chem. Biol. Interact. 178, 99–109. doi: 10.1016/j.cbi.2008.10.058 19061874

[B8] Breitling R. Marijanovi Z. Perovi D. Adamski J. (2001). Evolution of 17β-HSD type 4, a multifunctional protein of β-oxidation. Mol. Cell. Endocrinol. 171, 205–210. doi: 10.1016/S0303-7207(00)00415-9 11165031

[B9] Chen C. Chen H. Zhang Y. Thomas H. R. Frank M. H. He Y.. (2020). TBtools: an integrative toolkit developed for interactive analyses of big biological data. Mol. Plant 13, 1194–1202. doi: 10.1016/j.molp.2020.06.009 32585190

[B10] Czechowski T. Forestier E. Swamidatta S. H. Gilday A. D. Cording A. Larson T. R.. (2022). Gene discovery and virus-induced gene silencing reveal branched pathways to major classes of bioactive diterpenoids in euphorbia peplus. Proc. Natl. Acad. Sci. U.S.A. 119, e2203890119. doi: 10.1073/PNAS.2203890119 35584121 PMC9173813

[B11] Das A. K. Uhler M. D. Hajra A. K. (2000). Molecular cloning and expression of mammalian peroxisomal trans-2-enoyl-coenzyme a reductase cDNAs. J. Biol. Chem. 275, 24333–24340. doi: 10.1074/jbc.M001168200 10811639

[B12] Del Río L. A. López-Huertas E. (2016). ROS generation in peroxisomes and its role in cell signaling. Plant Cell Physiol. 57, 1364–1376. doi: 10.1093/pcp/pcw076 27081099

[B13] Deng Q. Li H. Feng Y. Xu R. Li W. Zhu R.. (2022). Defining upstream enhancing and inhibiting sequence patterns for plant peroxisome targeting signal type 1 using large-scale in silico and *in vivo* analyses. Plant J. 111, 567–582. doi: 10.1111/tpj.15840 35603488 PMC9542071

[B14] Endo S. Maeda S. Matsunaga T. Dhagat U. El-Kabbani O. Tanaka N.. (2009). Molecular determinants for the stereospecific reduction of 3-ketosteroids and reactivity towards all-trans-retinal of a short-chain dehydrogenase/reductase (DHRS4). Arch. Biochem. Biophys. 481, 183–190. doi: 10.1016/j.abb.2008.11.014 19056333

[B15] Eubel H. Meyer E. H. Taylor N. L. Bussell J. D. O’Toole N. Heazlewood J. L.. (2008). Novel proteins, putative membrane transporters, and an integrated metabolic network are revealed by quantitative proteomic analysis of arabidopsis cell culture peroxisomes. Plant Physiol. 148, 1809–1829. doi: 10.1104/pp.108.129999 18931141 PMC2593673

[B16] Fransen M. Van Veldhoven P. P. Subramani S. (1999). Identification of peroxisomal proteins by using M13 phage protein VI phage display: molecular evidence that mammalian peroxisomes contain a 2,4-dienoyl-CoA reductase. Biochem. J. 340, 561–568. doi: 10.1042/0264-6021:3400561 10333503 PMC1220285

[B17] Gloerich J. Ruiter J. P. N. Van Den Brink D. M. Ofman R. Ferdinandusse S. Wanders R. J. A. (2006). Peroxisomal trans-2-enoyl-CoA reductase is involved in phytol degradation. FEBS Lett. 580, 2092–2096. doi: 10.1016/j.febslet.2006.03.011 16546181

[B18] Gröger H. Hummel W. Metzner R. (2012). doi: 10.1016/B978-0-08-095167-6.00712-6

[B19] Gurvitz A. Rottensteiner H. Kilpeläinen S. H. Hartig A. Hiltunen J. K. Binder M.. (1997). The saccharomyces cerevisiae peroxisomal 2,4-dienoyl-coA reductase is encoded by the oleate-inducible gene SPS19. J. Biol. Chem. 272, 22140–22147. doi: 10.1074/jbc.272.35.22140 9268358

[B20] Hamid N. A. A. Zainal Z. Ismail I. (2018). Two members of unassigned type of short-chain dehydrogenase/ reductase superfamily (Sdr) isolated from persicaria minor show response towards aba and drought stress. J. Plant Biochem. Biotechnol. 27, 260–271. doi: 10.1007/s13562-017-0436-4

[B21] Han A. Xu R. Liu Y. Yin X. Lin Z. Yang W. (2021). HSDL2 acts as a promoter in pancreatic cancer by regulating cell proliferation and lipid metabolism. Onco. Targets. Ther. 14, 435–444. doi: 10.2147/OTT.S287722 33488098 PMC7814248

[B22] He X. Y. Shoukry K. Chu S. H. Yang J. J. Sprecher H. Schulz H. (1995). Peroxisomes contain Δ3,5,Δ2,4-Dienoyl-CoA isomerase and thus possess all enzymes required for the β-oxidation of unsaturated fatty acids by a novel reductase-dependent pathway. Biochem. Biophys. Res. Commun. 215, 15–22. doi: 10.1006/bbrc.1995.2428 7575583

[B23] Hofgen R. Willmitzer L. (1988). Storage of competent cells for agrobacterium transformation nucleic acids research rainer hofgen and lothar willmitzer. Nucleic Acids Res. 16, 9877. doi: 10.1093/nar/16.20.9877 3186459 PMC338805

[B24] Hua T. Wu D. Ding W. Wang J. Shaw N. Liu Z. J. (2012). Studies of human 2,4-dienoyl CoA reductase shed new light on peroxisomal β-oxidation of unsaturated fatty acids. J. Biol. Chem. 287, 28956–28965. doi: 10.1074/jbc.M112.385351 22745130 PMC3436514

[B25] Huang X. Q. Li R. Fu J. Dudareva N. (2022). A peroxisomal heterodimeric enzyme is involved in benzaldehyde synthesis in plants. Nat. Commun. 13, 1352. doi: 10.1038/s41467-022-28978-2 35292635 PMC8924275

[B26] Hyong W. C. Byung G. L. Nak H. K. Park Y. Chae W. L. Hyun K. S.. (2008). A role for a menthone reductase in resistance against microbial pathogens in plants. Plant Physiol. 148, 383–401. doi: 10.1104/pp.108.119461 18599651 PMC2528125

[B27] Jin Y. Zhang C. Liu W. Tang Y. Qi H. Chen H.. (2016). The the alcohol dehydrogenase gene family in melon (Cucumis melo l.): bioinformatic analysis and expression patterns. Front. Plant Sci. 7. doi: 10.3389/fpls.2016.00670 PMC4870255 27242871

[B28] Kallberg Y. Oppermann U. Jörnvall H. Persson B. (2009). Short-chain dehydrogenase/reductase (SDR) relationships: a large family with eight clusters common to human, animal, and plant genomes. Protein Sci. 11, 636–641. doi: 10.1110/ps.26902 PMC2373483 11847285

[B29] Klepikova A. V. Kasianov A. S. Gerasimov E. S. Logacheva M. D. Penin A. A. (2016). A high resolution map of the arabidopsis thaliana developmental transcriptome based on RNA-seq profiling. Plant J. 88, 1058–1070. doi: 10.1111/tpj.13312 27549386

[B30] Kowalik D. Haller F. Adamski J. Moeller G. (2009). In search for function of two human orphan SDR enzymes: hydroxysteroid dehydrogenase like 2 (HSDL2) and short-chain dehydrogenase/reductase-orphan (SDR-O). J. Steroid Biochem. Mol. Biol. 117, 117–124. doi: 10.1016/j.jsbmb.2009.08.001 19703561

[B31] Kramm A. Kisiela M. Schulz R. Maser E. (2012). Short-chain dehydrogenases/reductases in cyanobacteria. FEBS J. 279, 1030–1043. doi: 10.1111/j.1742-4658.2012.08494.x 22251568

[B32] Kumar S. Stecher G. Li M. Knyaz C. Tamura K. (2018). MEGA X: molecular evolutionary genetics analysis across computing platforms. Mol. Biol. Evol. 35, 1547–1549. doi: 10.1093/molbev/msy096 29722887 PMC5967553

[B33] Kunze M. (2020). The type-2 peroxisomal targeting signal. Biochim. Biophys. Acta - Mol. Cell Res. 1867, 118609. doi: 10.1016/j.bbamcr.2019.118609 31751594

[B34] Lei Z. Chen W. Zhang M. Napoli J. L. (2003). Reduction of all-trans-retinal in the mouse liver peroxisome fraction by the short-chain dehydrogenase/reductase RRD: induction by the PPARα ligand clofibrate. Biochemistry 42, 4190–4196. doi: 10.1021/bi026948i 12680773

[B35] Ma X. Liu Y. G. (2016). CRISPR/Cas9-based multiplex genome editing in monocot and dicot plants. Curr. Protoc. Mol. Biol. 2016, 1–21. doi: 10.1002/cpmb.10 27366892

[B36] Manríquez D. El-Sharkawy I. Flores F. B. El-Yahyaoui F. Regad F. Bouzayen M.. (2006). Two highly divergent alcohol dehydrogenases of melon exhibit fruit ripening-specific expression and distinct biochemical characteristics. Plant Mol. Biol. 61, 675–685. doi: 10.1007/s11103-006-0040-9 16897483

[B37] Matsunaga T. Endo S. Maeda S. Ishikura S. Tajima K. Tanaka N.. (2008). Characterization of human DHRS4: an inducible short-chain dehydrogenase/reductase enzyme with 3β-hydroxysteroid dehydrogenase activity. Arch. Biochem. Biophys. 477, 339–347. doi: 10.1016/j.abb.2008.06.002 18571493

[B38] Moeller G. Adamski J. (2006). Multifunctionality of human 17β-hydroxysteroid dehydrogenases. Mol. Cell. Endocrinol. 248, 47–55. doi: 10.1016/j.mce.2005.11.031 16413960

[B39] Möller G. Lüders J. Markus M. Husen B. Van Veldhoven P. P. Adamski J. (1999). Peroxisome targeting of porcine 17β-hydroxysteroid dehydrogenase type IV/D-specific multifunctional protein 2 is mediated by its c-terminal tripeptide AKI. J. Cell. Biochem. 73, 70–78. doi: 10.1002/(SICI)1097-4644(19990401)73:1<70::AID-JCB8>3.0.CO;2-K 10088725

[B40] Möller G. Van Grunsven E. G. Wanders R. J. A. Adamski J. (2001). Molecular basis of d-bifunctional protein deficiency. Mol. Cell. Endocrinol. 171, 61–70. doi: 10.1016/S0303-7207(00)00388-9 11165012

[B41] Moummou H. Kallberg Y. Tonfack L. B. Persson B. van der Rest B. (2012). The plant short-chain dehydrogenase (SDR) superfamily: genome-wide inventory and diversification patterns. BMC Plant Biol. 12, 219. doi: 10.1186/1471-2229-12-219 23167570 PMC3541173

[B42] Nakagawa T. Suzuki T. Murata S. Nakamura S. Hino T. Maeo K.. (2007). Improved gateway binary vectors: high-performance vectors for creation of fusion constructs in transgenic analysis of plants. Biosci. Biotechnol. Biochem. 71, 2095–2100. doi: 10.1271/bbb.70216 17690442

[B43] Ohlrogge J. B. Jaworski J. G. (1997). Regulation of fatty acid synthesis. Annu. Rev. Plant Biol. 48, 109–136. doi: 10.1146/annurev.arplant.48.1.109 15012259

[B44] Okamoto S. Yu F. Harada H. Okajima T. Hattan J. I. Misawa N.. (2011). A short-chain dehydrogenase involved in terpene metabolism from zingiber zerumbet. FEBS J. 278, 2892–2900. doi: 10.1111/j.1742-4658.2011.08211.x 21668645

[B45] Pan R. Hu J. (2018). Proteome of plant peroxisomes. Subcell. Biochem. 89, 3–45. doi: 10.1007/978-981-13-2233-4 30378017

[B46] Pan R. Kaur N. Hu J. (2014). The arabidopsis mitochondrial membrane-bound ubiquitin protease UBP27 contributes to mitochondrial morphogenesis. Plant J. 78, 1047–1059. doi: 10.1111/tpj.12532 24707813

[B47] Pan R. Liu J. Wang S. Hu J. (2020). Peroxisomes: versatile organelles with diverse roles in plants. New Phytol. 225, 1410–1427. doi: 10.1111/nph.16134 31442305

[B48] Pan R. Reumann S. Lisik P. Tietz S. Olsen L. J. Hu J. (2018). Proteome analysis of peroxisomes from dark-treated senescent arabidopsis leaves. J. Integr. Plant Biol. 60, 1028–1050. doi: 10.1111/jipb.12670 29877633

[B49] Persson B. Kallberg Y. (2013). Classification and nomenclature of the superfamily of short-chain dehydrogenases/reductases (SDRs). Chem. Biol. Interact. 202, 111–115. doi: 10.1016/j.cbi.2012.11.009 23200746

[B50] Poutanen M. Isomaa V. Peltoketo H. Vihko R. (1995). Role of 17β-hydroxysteroid dehydrogenase type 1 in endocrine and intracrine estradiol biosynthesis. J. Steroid Biochem. Mol. Biol. 55, 525–532. doi: 10.1016/0960-0760(95)00201-4 8547177

[B51] Quan S. Yang P. Cassin-Ross G. Kaur N. Switzenberg R. Aung K.. (2013). Proteome analysis of peroxisomes from etiolated arabidopsis seedlings identifies a peroxisomal protease involved in β-oxidation and development. Plant Physiol. 163, 1518–1538. doi: 10.1104/pp.113.223453 24130194 PMC3850190

[B52] Reumann S. Babujee L. Ma C. Wienkoop S. Siemsen T. Antonicelli G. E.. (2007). Proteome analysis of arabidopsis leaf peroxisomes reveals novel targeting peptides, metabolic pathways, and defense mechanisms. Plant Cell 19, 3170–3193. doi: 10.1105/tpc.107.050989 17951448 PMC2174697

[B53] Reumann S. Chowdhary G. (2018). Prediction of peroxisomal matrix proteins in plants. Subcell. Biochem. 89, 125–138. doi: 10.1007/978-981-13-2233-4_5 30378021

[B54] Reumann S. Quan S. Aung K. Yang P. Manandhar-Shrestha K. Holbrook D.. (2009). In-depth proteome analysis of arabidopsis leaf peroxisomes combined with *in vivo* subcellular targeting verification indicates novel metabolic and regulatory functions of peroxisomes1. Plant Physiol. 150, 125–143. doi: 10.1104/pp.109.137703 19329564 PMC2675712

[B55] Sato Y. Morita R. Katsuma S. Nishimura M. Tanaka A. Kusaba M. (2009). Two short-chain dehydrogenase/reductases, NON-YELLOW COLORING 1 and NYC1-LIKE, are required for chlorophyll b and light-harvesting complex II degradation during senescence in rice. Plant J. 57, 120–131. doi: 10.1111/j.1365-313X.2008.03670.x 18778405

[B56] Skogsberg J. Lundström J. Kovacs A. Nilsson R. Noori P. Maleki S.. (2008). Transcriptional profiling uncovers a network of cholesterol-responsive atherosclerosis target genes. PLoS Genet. 4, e1000036. doi: 10.1371/journal.pgen.1000036 18369455 PMC2265530

[B57] Su T. Shao Q. Wang P. Ma C. (2016). Oxidative stress and its role in peroxisome homeostasis in plants. Redox State as Cent. Regul. Plant-Cell Stress Responses, ed. Dharmendra F. J. C. Palma J. M. (Springer Cham), 117–136. doi: 10.1007/978-3-319-44081-1_6/FIGURES/2

[B58] Tarafdar S. Chowdhary G. (2022). Translating the arabidopsis thaliana peroxisome proteome insights to solanum lycopersicum: consensus versus diversity. Front. Cell Dev. Biol. 10. doi: 10.3389/fcell.2022.909604 PMC9328179 35912119

[B59] Thumuluri V. Armenteros J. A. Johansen A. R. Nielsen H. Winther O. (2022). DeepLoc 2.0: multi-label subcellular localization prediction using protein language models 50, 1–7. doi: 10.1093/nar/gkac278 PMC9252801 35489069

[B60] Wang X. Zhou R. Louie G. V. Mühlemann J. K. Bomati E. K. Bowman M. E.. (2014). Structural studies of cinnamoyl-CoA reductase and cinnamyl-alcohol dehydrogenase, key enzymes of monolignol biosynthesis. Plant Cell 26, 3709–3727. doi: 10.1105/tpc.114.127399 25217505 PMC4213152

[B61] Wiszniewski A. A. G. Zhou W. Smith S. M. Bussell J. D. (2009). Identification of two arabidopsis genes encoding a peroxisomal oxidoreductase-like protein and an acyl-CoA synthetase-like protein that are required for responses to pro-auxins. Plant Mol. Biol. 69, 503–515. doi: 10.1007/s11103-008-9431-4 19043666

[B62] Yu S. Sun Q. Wu J. Zhao P. Sun Y. Guo Z. (2021). Genome-wide identification and characterization of short-chain dehydrogenase/reductase (SDR) gene family in medicago truncatula. Int. J. Mol. Sci. 22, 9498. doi: 10.3390/ijms22179498 34502406 PMC8430790

[B63] Zhang S. Xie L. Zheng S. Lu B. Tao W. Wang X.. (2021). Identification, expression and evolution of short-chain dehydrogenases/reductases in nile tilapia (Oreochromis niloticus). Int. J. Mol. Sci. 22, 4201. doi: 10.3390/ijms22084201 33919636 PMC8073704

[B64] Ziegler J. Voigtländer S. Schmidt J. Kramell R. Miersch O. Ammer C.. (2006). Comparative transcript and alkaloid profiling in papaver species identifies a short chain dehydrogenase/reductase involved in morphine biosynthesis. Plant J. 48, 177–192. doi: 10.1111/j.1365-313X.2006.02860.x 16968522

[B65] Zolman B. K. Martinez N. Millius A. Adham A. R. Bartel B. (2008). Identification and characterization of arabidopsis indole-3-butyric acid response mutants defective in novel peroxisomal enzymes. Genetics 180, 237–251. doi: 10.1534/genetics.108.090399 18725356 PMC2535678

[B66] Zolman B. K. Nyberg M. Bartel B. (2007). IBR3, a novel peroxisomal acyl-CoA dehydrogenase-like protein required for indole-3-butyric acid response. Plant Mol. Biol. 64, 59–72. doi: 10.1007/s11103-007-9134-2 17277896

