# Supplementary figures and images for: Bioinformatic analysis of short-chain dehydrogenase/reductase proteins in plant peroxisomes
Source: Front Plant Sci. 2023 Jun 9;14:1180647. doi: 10.3389/fpls.2023.1180647 (PMC10288848; doi:10.3389/fpls.2023.1180647)

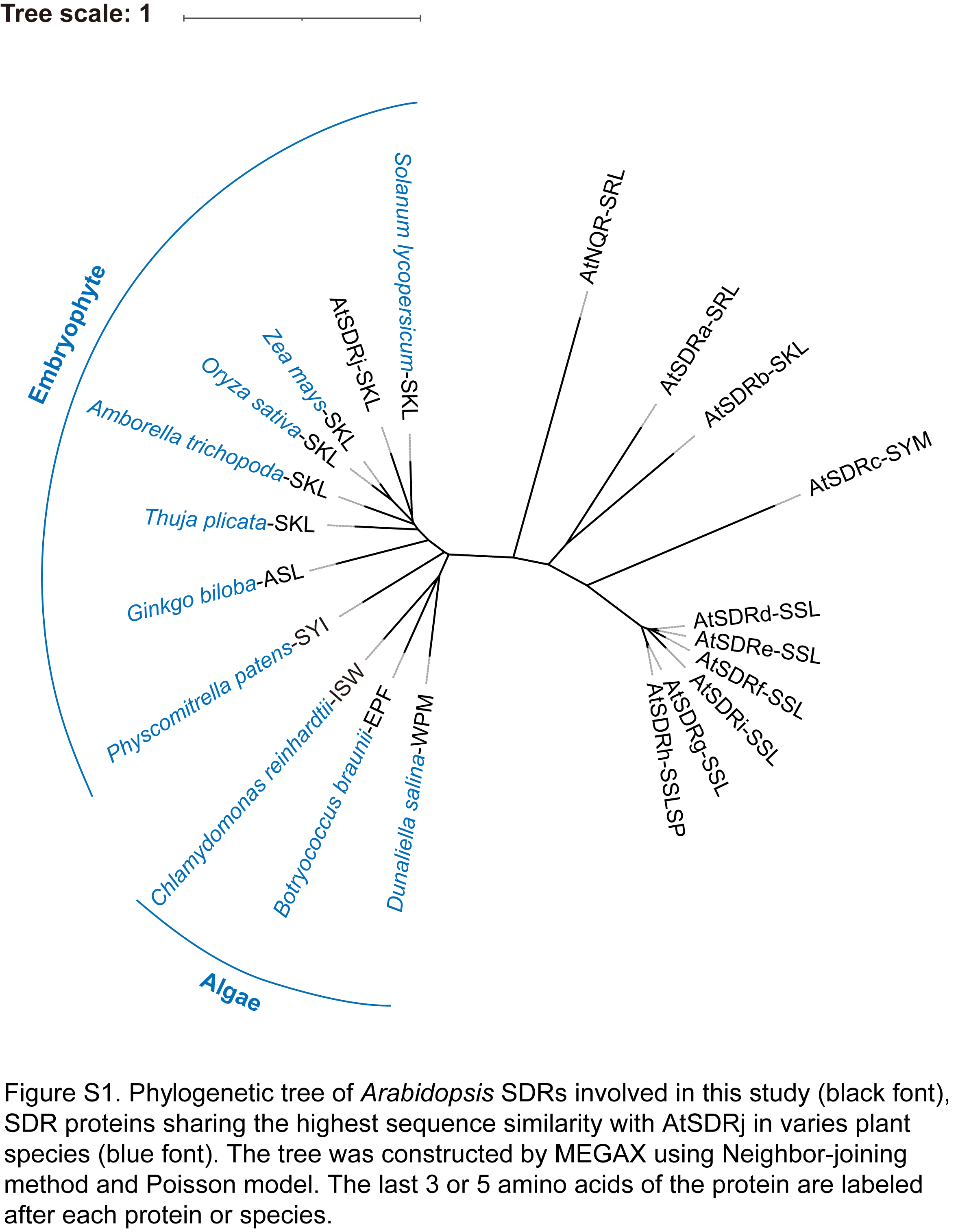

Supplement: Supplementary file 1 [file Image_1.tif]

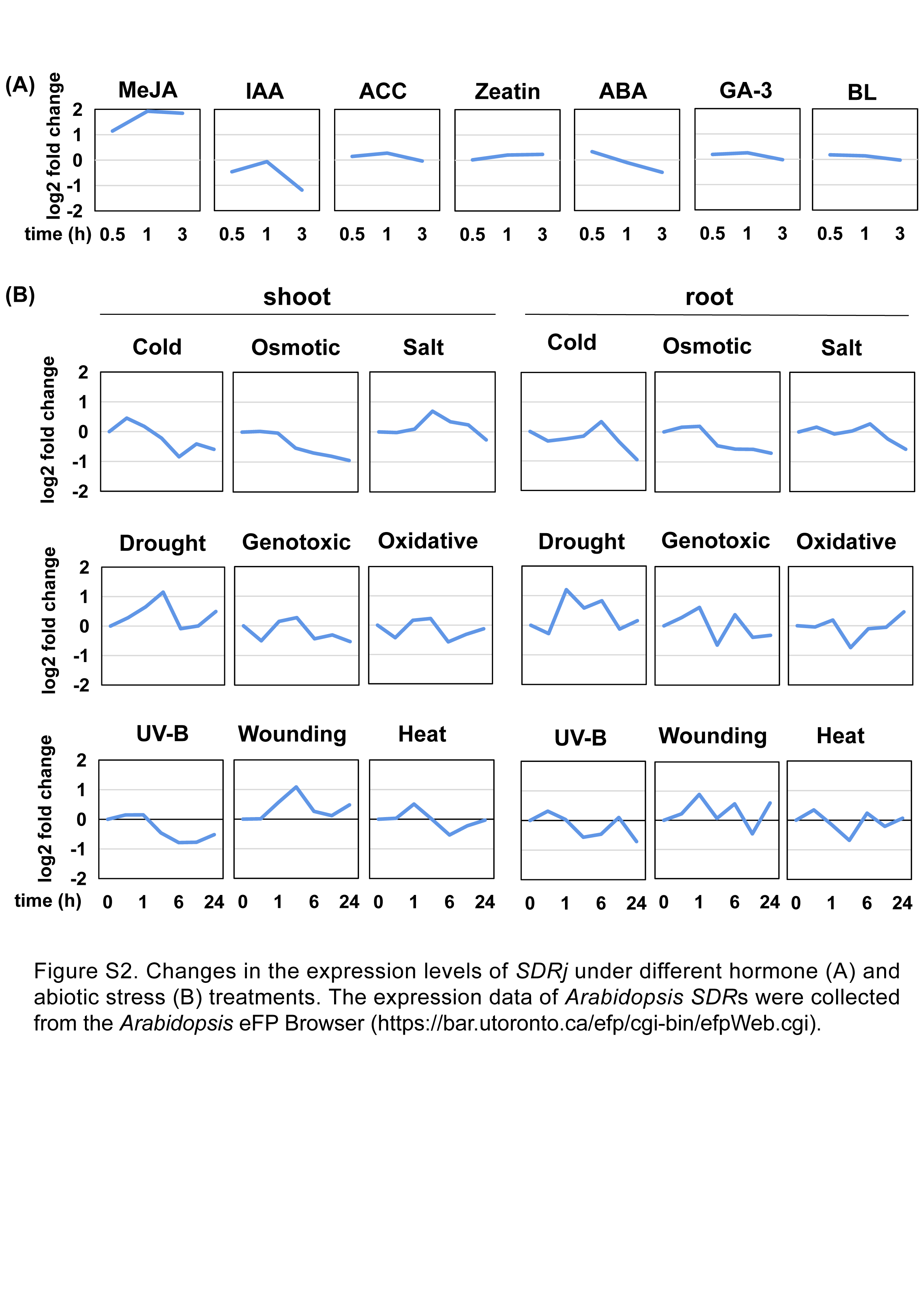

Supplement: Supplementary file 2 [file Image_2.tif]
